# Event and Comment

**Published:** 1923-06

**Authors:** 


### DENTAL REGISTER

THE MAGAZINE OF DENTISTRY

Vol. LXXVII | JUNE, 1923 | No. 6

### EVENT AND COMMENT

BY VOTRE AMI DENTAIRE

One of the principal ideas brought out at the commencement exercises of the Ohio College of Dental Surgery was to stick to the trail. One starts out with high ideals and great ambition and is soon beset with many cross and counter ideas and lures which require steadfastness on one's part to stick to the original ideal and make way to the desired goal of one's ambition. It is not easy to stick to the trail but it is only those who stick to the trail that reach the end of the trail, the place where the trail leads to—so it stands to reason that the cross trails, the alluring byways, requiring resistance sometimes, must be avoided if the end of the trail one starts on is to be reached. This idea applies to Dentistry as well as to any other vocation in life.

